# Uptake and linkage into care over one year of providing HIV testing and counselling through community and health facility testing modalities in urban informal settlement of Kibera, Nairobi Kenya

**DOI:** 10.1186/s12889-016-3033-x

**Published:** 2016-05-04

**Authors:** Samuel Muhula, Peter Memiah, Lilian Mbau, Happiness Oruko, Bebora Baker, Geoffrey Ikiara, Margaret Mungai, Meshack Ndirangu, Dunstan Achwoka, Festus Ilako

**Affiliations:** Amref Health Africa in Kenya, P.O Box 30125–00100, Nairobi, Kenya; University of West Florida, College of Health, 11000 University Parkway, Building 38, Room #131, Pensacola, Fl.32514 USA; Division of Global HIV/AIDS (DGHA), US Centers for Disease Control and Prevention, Village Market, P.O. Box 606–00621, Nairobi, Kenya; Amref Health Africa Headquarters, P.O Box 27691–00506, Nairobi, Kenya

**Keywords:** Health facility-based testing, Community-based testing, Kibera, Kenya

## Abstract

**Background:**

We examine the uptake of HIV Testing and Counselling (HTC) and linkage into care over one year of providing HTC through community and health facility testing modalities among people living in Kibera informal urban settlement in Nairobi Kenya.

**Methods:**

We analyzed program data on health facility-based HIV testing and counselling and community- based testing and counselling approaches for the period starting October 2013 to September 2014. Univariate and bivariate analysis methods were used to compare the two approaches with regard to uptake of HTC and subsequent linkage to care. The exact Confidence Intervals (CI) to the proportions were approximated using simple normal approximation to binomial distribution method.

**Results:**

Majority of the 18,591 clients were tested through health facility-based testing approaches 72.5 % (*n* = 13485) vs those tested through community-based testing comprised 27.5 % (*n* = 5106). More clients tested at health facilities were reached through Provider Initiated Testing and Counselling PITC 81.7 % (*n* = 11015) while 18.3 % were reached through Voluntary Counselling and Testing (VCT)/Client Initiated Testing and Counselling (CITC) services. All clients who tested positive during health facility-based testing were successfully linked to care either at the project sites or sites of client choice while not all who tested positive during community based testing were linked to care. The HIV prevalence among all those who were tested for HIV in the program was 5.2 % (*n* = 52, 95 % CI: 3.9 %–6.8 %). Key study limitation included use of aggregate data to report uptake of HTC through the two testing approaches and not being able to estimate the population in the catchment area likely to test for HIV.

**Conclusion:**

Health facility-based HTC approach achieved more clients tested for HIV, and this method also resulted in identifying greater numbers of people who were HIV positive in Kibera slum within one year period of testing for HIV compared to community-based HTC approach. Linking HIV positive clients to care proved much easier during health facility- based HTC compared to community- based HTC.

## Background

HIV continues to be a major global public health issue having claimed more than 39 million lives [1]. Sub-Saharan Africa continues to be the most affected region with the HIV epidemic, accounting for about 71 % (24.7 million) of people living with HIV globally [2]. In 2013, 71 % of the 2.1 million global new infections and 73 % of the 1.5 million HIV related deaths occurred in the region [1]. Despite major investments in HIV testing, treatment, and prevention programmes, only one quarter of adult Africans have had a recent HIV test, and half of people living with HIV in Sub-Saharan Africa do not know they are HIV positive [3].

In the early days of HIV response, VCT was the conventional way through which persons learnt their HIV status and was adopted in 1980s [4]. The approach mainly stresses the need for persons to voluntarily give information and have informed consent for the ethical conduct of HTC [5]. Millions of people have become aware of their HIV status through VCT and further linked to HIV prevention and care services. In an effort to expand access to prevention and care amenities, World Health Organization (WHO) guidelines recommend a combination of strategically selected delivery models to provide HTC. Such models include community-based HTC, facility HTC, self-testing, work-place testing and couple testing. No solitary delivery model will serve all who could benefit from HTC in a given setting or country [4].

### HIV/AIDS context in Kenya

As of 2012, about 1.19 million adult (15 to 64 years) Kenyans were living with HIV infection, translating to a prevalence of 5.6 % [6]. According to Kenya Demographic Health Survey 2014, 8.3 % of urban residents engaged in sexual intercourse with two or more sexual partners 12 months prior to the survey compared to 6.2 % rural residents who reported the same [7]. This means that urban residents in Kenya increase their chances of contracting and transmitting HIV by engaging in sexual intercourse with multiple partners compared to rural residents. However, even in urban areas there are massive differences in HIV prevalence with urban slum settlements having a significantly higher prevalence of HIV at 12 % [8] than the corresponding prevalence in non-slum urban (6.5 %) and rural (5.1 %) areas [6, 7]. This is much higher than the national average of 5.6 % and the overall prevalence in Nairobi of 4.9 % [6].

Urban poverty and the high mobility of the urban poor may increase vulnerability to HIV/AIDS [9]. Poverty may increase vulnerability to HIV through several pathways including: a greater likelihood to engage in transactional sex; limited access to formal education and skills, which heightens economic dependency especially for women and girls; and limited access to HIV preventive services and health information [10]. On the other hand, high mobility may increase vulnerability to HIV through increased access to casual sexual partners and weakened social controls [11]. Studies conducted in Kenya show that adolescent slum dwellers show stronger sexual resilience in households headed by fathers [12].

### HIV testing and counselling in Kenya

Kenya Demographic Health Survey 2014 indicate that 91 % of adults in Kenya know where to take an HIV test, 85 % have ever tested for HIV while 53 % have tested for HIV in the past 12 months and received results of the latest test. The proportions of young people aged 15–24 years in Nairobi with knowledge of HIV prevention methods are women 63 % and men 71 % while the percentages of women and men in Nairobi who have not been tested for HIV in the past 12 months and received the results of the last test stand at 40 and 42 % respectively [7]. The 9 % of adults who do not know where to take an HIV test, 15 % who have never tested for HIV and, 47 % who have not tested for HIV in the past 12 months, need effective approaches to be tested for the country to be able to achieve close to 100 % coverage of HIV testing. Health education especially to the youth is equally important to improve aspects such as knowledge of HIV prevention methods. Those who are HIV infected will use the knowledge of their HIV status to take action to protect their sexual partners, access treatment and plan for the future.

Amref Health Africa in Kenya, through funding from US Centre for Disease Control and Prevention, implemented a care and treatment project within Kibera informal settlement (one of the largest informal settlements in Kenya) starting in 2005 and to end in 2016. The project focused on expansion of High Quality HIV Prevention, Care and Treatment activities at facility and community levels including HTC to expand coverage of services in Kibera Slum of Nairobi. We therefore compare among those counselled and tested within a period of one year at the project, the proportions who take up HIV testing through community based testing approaches verses health facility based testing approaches and as well look at linkage into care of clients who test HIV positive during the two testing approaches. We do this by describing the characteristics of people accessing HTC through the two testing modalities and demonstrate any preferred approaches. The findings will be of great significance to HIV prevention stakeholders in terms of designing relevant prevention strategies to urban informal settings such as Kibera slum.

## Methods

### Study design and procedures

Kibera Care and Treatment project is implemented in 4 health centers within Kibera informal settlement. The data used in this study was collected using registers that captured client routine visits at the four health centers and during outreach activities within the facility catchment areas. Data recording in the registers was done at the point of care by the various service providers. The two testing approached used are 1) *Health Facility-Based HIV Testing and Counselling* is defined as HTC offered in clinical settings to help in clinical management and is commonly offered through client-initiated voluntary HIV counselling and testing (VCT/CITC) and provider-initiated testing and counselling (PITC) [4]. Nurses at the four health facilities provided HTC services through VCT/CITC, PITC, Couple counselling and testing, pediatric testing which involves educating mothers on family testing and screening of HIV Exposed Infants (HEI). Trained CHVs provided health education at outpatient waiting bays at the four health facilities. The facility based testing services were available all through at the 4 health facilities and were provided using MOH HIV testing algorithm. 2) *Community-based testing and counselling* services are defined as HTC outside of health facilities and are likely to build public confidence, protect human rights and lessen stigma and discrimination [4]. This program implemented five different community-based HTC approaches namely; home based testing and counselling, evening hours testing, weekend testing, church services testing and testing during outreach services where other services such as health education were offered. A joint team of nurses and Community Health Volunteers (CHVs) conducted community based HTC. The team used similar HIV testing algorithm as used in health facility HTC. Home based testing and counselling was done through door-to-door home visits by a team of nurses and CHVs at least once a month within the Kibera informal settlement. Outreach services were conducted once a quarter and preceded by a whole day of community mobilization, two days before the outreach services event. Outreach services were organized by pitching tents in open fields, with loud public address system, entertainment, health education while testing services ongoing at the tents. Evening hours testing and weekend testing were organized by pitching tents at strategic locations within Kibera informal settlement and CHVs tasked with informing the public of the availability of the services in their neighborhoods. Church services testing were conducted on Sundays within church compounds, specifically targeting members at the end of the church service. The availability of the testing services was announced before end of the church service and CHVs tasked with directing the public to the testing tents.

### Study setting and participants

The study took place within Kibera informal settlement. Depending on the source, the population figures of Kibera slum continue to vary, with UN-HABITAT estimating the population at not more than 900,000 individuals [13]. The area is characterized by inadequate access to formal health, safe water, sanitation and other infrastructure; poor structural quality of housing; overcrowding; insecure residential status and poor health indicators. Lack of proper health services and facilities is a major problem in the slum caused by inadequate support by the government and other stakeholders. The collective effects of inadequate health services, poverty, and difficult socio-environmental conditions increase slum dwellers’ vulnerability to poor health outcomes [14, 15].

The study participants were all children and adults of all ages who underwent testing through any of the two modalities within Kibera informal settlement during the one year period.

### Data collection, processing and analysis

Monitoring data collected using the Ministry of Health data collection registers for the period October 2013 to September 2014 were used in this study. Data collected included demographic information, HIV testing approaches, first testing or repeat testing clients, their attendance as individuals or couples, HIV status (positive, negative), linkage into care and treatment and HTC uptake among the two testing modalities. The data collected using the registers were summarized in monthly reporting tools before being entered into DHIS2 system which is a central Ministry of Health reporting platform. Data used in this study was then accessed from DHIS2 system for analysis. Clients considered to have been linked into care and treatment were those who presented at the project four health facilities with a referral form from a CHV and documented in pre-ART register after CD4 count is done. Clients who reported attending care at other health facilities were also considered linked into care after confirmation with the health facility through calling. HTC uptake in this study is the proportion of clients who take up HTC through each of the two testing modalities (community or facility based) and recorded in Ministry of Health registers over the total number who were tested in the program. Reported data in DHIS2 system was examined for completeness and then exported to MS excel where univariate and bivariate analysis of the data were conducted by generating cross tabulations to determine the relationship between variables under comparison. The exact Confidence Intervals (CI) were approximated using simple normal approximation to binomial distributions method.

## Results

A total of 18,591 clients were tested for HIV during the period under study. Majority were tested through health facility-based testing approaches 72.5 % (*n* = 13485) compared to those tested through community-based testing approach 27.5 % (*n* = 5106). In health facility testing, more clients were reached through PITC 81.7 % (*n* = 11015) while 18.3 % were reached through VCT/CITC services. Almost two-thirds (63.5 %) of all the clients tested for HIV were repeat testers (*n* = 11803) vs first time testers 36.5 % (*n* = 6788) (See Table 1).

**Table 1:** HIV testing and counselling of clients presenting as individuals

	PITC		VCT		Total	
Characteristics	n	Percentage (%)	n	Percentage (%)	n	Percentage (%)
HIV Testing Approach						
Health Facility Testing	11015	81.7 %	2470	18.3 %	13485	72.5 %
Community Based Testing					5106	27.5 %
Total	11015		2470		18591	
HTC stages						
First Testing					6788	36.5 %
Repeat Testing					11803	63.5 %
Total					18591	

At health facilities-based testing (see Table 2), most of the 86.1 % (*n* = 13485) clients tested were aged above 14 years of which female clients comprised 62.2 %. Young people (>0 years and ≤ 14 years) comprised 13.9 %.

**Table 2:** Health facility testing

	>0 and ≤14 years		>14 years		Total	
Characteristics	n	Percentage (%)	n	Percentage (%)	n	Percentage (%)
Clients Counselled and Tested						
Male	949	50.7 %	4385	37.8 %	5334	39.6 %
Female	923	49.3 %	7228	62.2 %	8151	60.4 %
Total	1872		11613		13485	
HIV Status						
Male HIV Prevalence	17	1.8 %	269	6.1 %	286	5.4 %
Female HIV Prevalence	20	2.2 %	587	8.1 %	607	7.4 %
Total	37	2.0 %	856	7.4 %	893	6.6 %
Linkage to care	37	100 %	856	100 %	893	100 %
General outpatient workload for New clients >5 years old					42593	
HTC Uptake at Health Facilities					13485	31.7 %

HIV prevalence among clients tested at health facilities was 6.6 % (*n* = 893) vs HIV prevalence at community based testing which was 1.3 % (*n* = 68) (see Table 3).

**Table 3:** Community based testing

	n	Percentage (%)
Community Based Testing	5106	
HIV Positive	68	1.3 %

All clients who tested positive during health facility-based testing were successfully linked to care either at the project sites or sites of client choice while not all who tested positive during community based testing were linked to care. At health facility testing, clients aged above 14 years were more likely to be HIV positive 7.4 % (*n* = 856) compared to the young people aged below or equal to 14 years whose HIV prevalence was 2.0 %. The HIV prevalence among all clients who were tested for HIV in the program was 5.2 % (*n* = 52, 95 % CI: 3.9 %–6.8 %) (see Table 4).

**Table 4:** HIV prevalence among those who were tested for HIV in the Program

	n	Percentage (%)	95 % CI
HIV Positive at Health Facility	893		
HIV Positive at Community Based Testing	68		
Total Tested at Community and Health Facility	18591		
HIV Prevalence		5.2 %	(3.9 % 6.8 %)

Health education at the outpatient unit focused on importance of clients knowing their HIV status and appropriately referred for testing after consenting. HTC uptake among new clients presenting at the clinics for outpatient services was 32 %. A total of 2444 couples were tested during facility and community-based testing of which 3.2 % turned out to be discordant couples (*n* = 78, 95 % CI: 2.5 %–4.0 %) vs 11.2 % who were concordant positive (*n* = 274, 95 % CI: 10 %–12.5 %) (see Table 5).

**Table 5:** Couples testing

	n	Percentage (%)	95 % CI
Couples Testing	2444		
Concordant HIV positive Couples	274	11.2 %	(10.0 % 12.5 %)
Discordant Couples	78	3.2 %	(2.5 % 4.0 %)
Total	352		

### Program constraints

One of the benefits of community-based testing, especially door-to-door testing, is allowing couples and families to be counselled about their HIV status, behavior change, ART, and prevention interventions together [16, 17]. This program reported challenges with convincing couples to get tested together. Although there was no evidence of any harm resulting from being tested in community-based HTC approaches, there were reports of fear of status disclosure or stigma, domestic violence among couples especially during discordancy. The program also faced difficulties linking HIV positive clients identified through community-based testing to care. In addition, community based testing were more expensive with high costs incurred organizing activities such as outreaches, door to door testing, yet few clients got tested with low positivity rate. The program faced acute shortages of test kits during the first quarter of the period leading to missed opportunities.

## Discussion

The high HIV prevalence among urban slum dwellers in Kenya [8] has motivated investigation and programmatic efforts to comprehend and address their sexual behavior. This study found out that the HIV prevalence among all clients who were tested for HIV in the program was 5.2 % which was higher than the overall prevalence in Nairobi of 4.9 % obtained through population survey, though not substantially different. This HIV prevalence is however significantly lower than urban slum settlements HIV prevalence of 12 %, which could be as a result of the different methodologies used in the two studies. In this study, HIV positivity rate among clients in community based HTC approaches was generally lower than among clients in facility-based HTC. Again, almost three quarters of clients who tested for HIV were tested in facility-based HTC. This could be because symptomatic people with HIV are more likely to visit health facilities, healthcare workers are more likely to offer HTC to patients with symptoms that might be associated with HIV. It could also be that fewer people were choosing to test in the community than in the facility and that those who do test in the community are less likely to be HIV positive than those who test in the facility. This is contrary to a randomized community-based HTC conducted in Tanzania, Zimbabwe and Thailand which found out a four-fold increase in the number of newly diagnosed people with HIV compared to standard clinic-based VCT [18], in which case PITC was not part of the study. A study in Zimbabwe found out that community-based HTC has the potential to decrease HIV stigma by normalizing HIV testing, and is an opportunity to provide prevention interventions for HIV and other diseases to asymptomatic populations [19]. Community based HTC turned out to be expensive in this project while in other contexts broadening community-based HTC to include preventive interventions and screening for other diseases could further improve cost-effectiveness [20].

Given that about 63 % of clients who went for HIV testing in this study were repeat testers show the widespread knowledge on the need to regularly test for HIV.

HTC uptake among new clients presenting at the clinics for outpatient services was low at 31.7 %, emphasizing the need to strengthen educational sessions at health facility outpatient waiting bays. While home based testing and counselling, testing during evening hours, weekend testing, testing at church services and testing during outreach services could be important approaches in some settings, the uptake of these approaches was lower than that of facility based approach. Further research on uptake of these approaches is important in informing future programing.

We observed substantial gender and age differences as factors associated with facility-based HIV testing among the residents of Kibera urban slum settlements. In particular, females are more likely to be tested for HIV at health facilities than males and adults were more likely to test for HIV than the younger clients (>0 years and ≤14 years). These results are not shocking. This pattern was similar to what was observed in KAIS 2012 [6] where in general female had higher HIV prevalence than male. Although more than 80 % of clients who tested for HIV at health facilities were reached through PITC approach, which shows that implementation of PITC as a standard component in clinical care decreases lost opportunities to testing for HIV. It is important to integrate both PITC and CITC/VCT to be able to completely minimize lost opportunities to testing for HIV. Linkage into care and treatment for clients tested HIV positive at health facility testing was at 100 %, highlighting successful referral systems and linkage structures put in place within the facilities to be able to provide immediate basic care support services to persons diagnosed with HIV. More needs to be done to be able to successfully link those whose results are positive during community based testing to care given that this was a great challenge in the programme.

### Study limitation

The main study limitations included relying on aggregate data reported in DHIS2 system rather than individual client data to report uptake of HTC and not being able to estimate the overall population in the project catchment area most likely to test for HIV.

## Conclusion

Health facility-based testing approach has proved to be an effective approach in reaching people for HIV testing leading to higher uptake within one year period of testing for HIV compared to community based testing approach in Kibera slum. Health facility-based testing approach is also better in identifying HIV positive individuals and in linking those identified as HIV positives into care and treatment.

## Ethical considerations

Ethical clearance was obtained from Kenya Medical Research Institute Ethical Review Committee and CDC Associate Director for Science to use the project database for research purposes. All patient encounter forms were filled and kept confidential in lockable cabinets within the clinic. Aggregate data used in this study was obtained from a central Ministry of Health reporting platform (DHIS2) hence needed no de-identification since no patient’s identifiers are reported into the system.

## Consent to Publish

Not applicable.

## Availability of data and materials

All the data supporting the findings of this study are available as presented in this paper.

## Abbreviations

CHVs: Community Health Volunteers
CI: Confidence Intervals
CITC: Client Initiated Testing and Counselling
HBTC: home based counselling and testing
HEI: HIV Exposed Infants
HTC: HIV testing and counselling
PITC: Provider Initiated Testing and Counselling
VCT: Voluntary Counselling and Testing

## References

[1] UNAIDS. Fact Sheet 2014: Global Statistics. 2014 [cited 2015 Jul 31]. Available from: http://www.unaids.org/sites/default/files/en/media/unaids/contentassets/documents/factsheet/2014/20140716_FactSheet_en.pdf.

[2] WHO. HIV/AIDS Fact Sheet. [cited 2015 Jul 31]. Available from: http://www.who.int/mediacentre/factsheets/fs360/en/.

[3] UNAIDS. Joint United Nations Programme on HIV/AIDS 2014. The gap report. Geneva. [cited 2015 Jun 10]. Available from: http://www.unaids.org/sites/default/files/en/media/unaids/contentassets/documents/unaidspublication/2014/UNAIDS_Gap_report_en.pdf.

[4] WHO. Service delivery approaches to HIV testing and counselling (HTC):A strategic policy framework. [cited 2015 Apr 21]. Available from: http://www.who.int/hiv/pub/vct/htc_framework/en/

[5] Bayer R, Edington C (2009). HIV testing, human rights, and global AIDS policy: exceptionalism and its discontents. J Health Polit Policy Law.

[6] National AIDS and STI Control Programme. [cited 2015 Jul 31] Kenya AIDS Indicator Survey 2012. Available from: http://www.nacc.or.ke/index.php/about-nacc/403-kais-2012-final-report.

[7] Kenya National Bureau of Statistics (2014). Kenya Demographic Health Survey 2014: Final Report.

[8] African Population and Health Research Center. Population and Health Dynamics in Nairobi’s Informal Settlements: Report of the Nairobi Cross-sectional Slums Survey (NCSS) 2012. [cited 2015 Jun 10]. Available from: http://aphrc.org/wp-content/uploads/2014/08/NCSS2-FINAL-Report.pdf.

[9] Dodoo FN-A, Zulu EM, Ezeh AC (2007). Urban–rural differences in the socioeconomic deprivation–Sexual behavior link in Kenya. Soc Sci Med.

[10] Krishnan S, Dunbar MS, Minnis AM, Medlin CA, Gerdts CE, Padian NS (2008). Poverty, gender inequities, and women’s risk of human immunodeficiency virus/AIDS. Ann N Y Acad Sci.

[11] Voeten HACM, Vissers DCJ, Gregson S, Zaba B, White RG, deVlas SJ (2010). Strong association between in-migration and HIV prevalence in urban Sub-Saharan Africa. Sex Transm Dis.

[12] Ngom P, Magadi MA, Owuor T (2003). Parental presence and adolescent reproductive health among the Nairobi urban poor. J Adolesc Health.

[13] UN-HABITAT. The State of African Cities 2010: Governance, Inequality and Urban Land Markets. [cited 2015 Apr 21]. Available from: https://www.citiesalliance.org/sites/citiesalliance.org/files/UNH_StateofAfricanCities_2010.pdf.

[14] Mark Montgomery M. Urban Poverty and Health in Developing Countries. Population Bulletin 64, no2. Jun 2009. [cited 2015 Apr 21]. Available from: http://www.igwg.org/pdf09/64.2urbanization.pdf.

[15] Rice J, Rice JS (2009). The concentration of disadvantage and the rise of an urban penalty: urban slum prevalence and the social production of health inequalities in the developing countries. Int J Health Serv Plan Adm Eval.

[16] WHO | Guidance on couples HIV testing and counselling - including antiretroviral therapy for treatment and prevention in serodiscordant couples. WHO. [cited 2015 May 28]. Available from: http://www.who.int/hiv/pub/guidelines/9789241501972/en/23700649

[17] WHO. Planning, implementing and monitoring home-based HIV testing and counselling. [cited 2015 May 28]. Available from: http://www.who.int/hiv/pub/vct/home_based_care/en/

[18] Sweat M, Morin S, Celentano D, Mulawa M, Singh B, Mbwambo J (2011). Increases in HIV Testing and Case Detection from NIMH Project Accept (HPTN 043) among 16–32 year olds: a randomized community-based intervention in Tanzania, Zimbabwe, and Thailand. Lancet Infect Dis.

[19] Chirawu P, Langhaug L, Mavhu W, Pascoe S, Dirawo J, Cowan F (2010). Acceptability and challenges of implementing voluntary counselling and testing (VCT) in rural Zimbabwe: evidence from the Regai Dzive Shiri Project. AIDS Care.

[20] Kahn JG, Muraguri N, Harris B, Lugada E, Clasen T, Grabowsky M (2012). Integrated HIV testing, malaria, and diarrhea prevention campaign in Kenya: modeled health impact and cost-effectiveness. PLoS One.

